# A Delay from Diagnosis to Treatment Is Associated with a Decreased Overall Survival for Patients with Endometrial Cancer

**DOI:** 10.3389/fonc.2016.00031

**Published:** 2016-02-12

**Authors:** Darren Dolly, Andreea Mihai, B. J. Rimel, Louis Fogg, Jacob Rotmensch, Alfred Guirguis, Edgardo Yordan, Summer Dewdney

**Affiliations:** ^1^Elkhart General Hospital, Elkhart, IN, USA; ^2^University of Iowa Hospitals and Clinics, Iowa City, IA, USA; ^3^Cedars-Sinai Medical Center, Los Angeles, CA, USA; ^4^Rush University Medical Center, Chicago, IL, USA

**Keywords:** endometrial cancer, disparities of care, delay in treatment, overall survival, insurance, health, race

## Abstract

**Objectives:**

While Caucasian women are more likely to be diagnosed with endometrial cancer compared to African-American women, the rate of mortality is higher for African Americans. The cause of this disparity is unknown. We analyzed the time interval from diagnosis of endometrial cancer to treatment as it pertains to race and socioeconomic factors and its possible impact on survival.

**Methods:**

This was a retrospective, single institution chart review using a cancer registry database. We identified 889 patients who were diagnosed with endometrial cancer between January 2005 and June 2012. Clinicopathologic characteristics, demographics, insurance status, distance from medical center, body mass index (BMI), dates of diagnosis, and treatment were obtained from the medical records. Survival and association was determined by a one-way ANOVA test.

**Results:**

At the time of the study, 699 patients were alive and 190 dead. The average age was noted to be 62 years (24–91 years). Stages I–IV disease accounted for 69, 6, 15, and 10%, respectively. White race accounted for 64%, African Americans 24%, and Hispanics 7% of our study population. Majority of patients were privately insured (*n* = 441) followed by Medicare (*n* = 375). The mean interval time from diagnosis to treatment was 47.5 days (0–363). A statistically significant difference was noted for this time interval with regard to both race and insurance status: white and African Americans (42.6 vs. 57.3 days, *p* = 0.048), privately insured and Medicare (38.4 vs. 54.1 days, *p* < 0.001). There was a significant association with increased risk of death with a longer delay (43.3 vs. 64.8 days, *p* < 0.001). No statistically significance was noted for distance from medical center or BMI.

**Conclusion:**

A significant increase in interval of time from diagnosis to treatment of endometrial cancer was seen in both race and insurance status. A longer interval from diagnosis to treatment was associated mortality. The causes of these delays are likely multifactorial but deem further investigation given these data.

## Introduction

According to National Cancer Institute Surveillance Epidemiology and End Results (SEER), it is estimated that 49,560 women were diagnosed and 8,190 women died from cancer of the uterus in 2013 (1). Endometrial cancer, which accounts for 95% of cancer of the uterine corpus, is the most common gynecologic malignancy (2). Racial differences in the incidence and mortality of endometrial cancer have been noted with higher incidence in Caucasian women compared to African-American women; however, the mortality rate is 85% higher for African-American women (1). The cause of this disparity in mortality rates among Caucasian women and African-American women is thought to be multifactorial. Some studies have shown that African-American women present with poor prognostic features, such as higher grade tumors (Grades II and III), advanced stage (Stages III or IV), and non-endometrioid (Type II) endometrial cancers (3). In one study by Setiawan et al., African Americans and Latinas had higher proportions of high-grade tumors (32.7 and 29.5%, respectively) compared to whites (19.2%) as well as more aggressive histology among African Americans (30.9%) and Latinas (26.2%) compared to whites (8.7%) (4). Other authors have suggested that the type of initial treatment offered to African-American women may have increased the mortality rate with Caucasian women being more likely than African Americans to receive surgery and radiation therapy (5). Another possible explanation is that the molecular phenotypes of endometrial cancers that arise in African-American women tend to have a higher rate of TP53 inactivation and decreased expression of PTEN (6). p53 tumor suppressor gene inactivation has been associated with more adverse histologies and advanced-stage disease, while PTEN mutation, the most frequent molecular alteration observed in endometrial cancer, is associated with a more favorable outcome (6).

Few studies have explored interval between diagnosis of endometrial cancer and the time of treatment. Minority races have been associated with lower socioeconomic status that may limit access to care. The objective of this study is to examine the interval from diagnosis to treatment in relation to race, socioeconomic status, and payor status at a single tertiary care institution.

## Materials and Methods

Using the Cancer Registry at Rush University Medical Center, we performed a retrospective chart review of patients diagnosed with and/or treated for endometrial cancer from January 1, 2005 to June 1, 2012. The Rush University Medical Center Internal Review Board approved this study. Patients were selected if they were diagnosed with primary cancer involving the uterus and were initially diagnosed and/or treated within the Rush University Medical Center network. Of note, all patients were treated by an attending gynecologic oncologist; there is no house staff clinic present at this institution. Patients whose primary tumor was outside the uterine corpus, who were diagnosed with primary cervical cancer or with uterine sarcomas, were excluded. The following information was extracted from patient charts: age at initial diagnosis, race/ethnicity, body mass index (BMI), insurance status, zip code, date of initial diagnosis, date of initial treatment, type of initial treatment (either surgical, radiation therapy, or chemotherapy), stage, histological type, and vital status (dead or alive) at the time of data collection. Time of diagnosis was determined from the date a pathological specimen was collected (day 0) either by endometrial sampling or from the initial surgery for a non-malignant cause (18.3%). For 20 cases, no treatment date was available and these patients were excluded from further analysis in this study. Date of treatment was determined using the date of patient’s surgical staging procedure or for patients who did not undergo surgery the initial date of radiation/chemotherapy treatment; for patients who underwent a hysterectomy for another cause (prior to diagnosis of endometrial cancer), the date of their hysterectomy was used. The interval treatment time was determined by the number of days between the dates of diagnosis and treatment. Distance from Rush University Medical Center was determined using patients’ listed home zip code and calculating the distance from that zip code against that of the medical center, this calculation was performed in the standard fashion. Analysis of the data was performed using the analytical software SPSS statistics 21.0; chi square test was used to analysis stage and vital status. For analysis of race and insurance status on treatment delay, we performed ANOVA. To see the relationship of BMI and distance from treatment center and its impact on delay in treatment time, we performed a regression analysis. A multivariate analysis was also performed.

## Results

A total of 964 charts were reviewed for this study. Seventy-five charts did not meet inclusion criteria leaving a total of 889 charts for analysis. Demographic information is outlined in Table 1. Average age of all patients was 62 years old (range 24–91 years). Of the cases reviewed, 64.3% were white, 24.3% were African-American, 7.0% were Hispanic, 0.7% Asian, and 3.6% were of other or unknown race. In terms of stage of disease at time of diagnoses, Stage I disease accounted for 68.8% (612/889), Stage II 6.4% (57/889), Stage III 14.5% (129/889), and Stage IV 10.1% (90/889); one patient stage was unknown. The majority of the histologic types were grades 1 and 2 endometrioid adenocarcinoma, 31.6% (281/889) and 30.3% (271/889), respectively. Poorly differentiated cancers made up 26.1% (232/889) including a combination of grade 3 endometrioid adenocarcinoma, serous, carcinosarcoma, and leiomyosarcoma. The remaining histologic group was defined in the registry as “cell type not determined” (11.8%). Majority of patients, 49.6% (441/889), had private insurance, followed closely by Medicare 42.1% (374/889) and Medicaid 4.4% (39/889). Average distance from health center was noted to be 23.5 miles (range 0–1,022 miles); for one patient, no information on zip code was available and thus unable to calculate distance. Average BMI of patients in study was 35 kg/m^2^; however, information was missing for 145 (16%) patients. At the time of data collection, 699 (78.6%) patients were alive and 190 (21.4%) were dead. As it would be expected, vital status varied between stages with majority of Stage I patient being alive at the time of analysis of this study. For stage I disease, 89.9% (550) patients were alive vs. 73.7% for stage II, 59.7% for stage III, and only 33.3% for stage IV. It is important to note that the cause of death was unknown and deaths include all causes for mortality. The mean interval from diagnosis to initial treatment was 47.9 days and ranged from 0 to 363 days. This interval when compared to survival and was noted to be statistically significant with patients who were still alive having a mean treatment interval of 43.35 days compared to those who were dead having a mean interval of 64.84 days (*p* < 0.001).

**Table 1:** **Demographic of patients**.

Demographics	Number of patients
**Race**	
White	572 (64.3%)
African-American	216 (24.3%)
Hispanic	62 (7%)
Asian	7 (0.7%)
Other/unknown	32 (3.6%)
**Stage of disease**	
Stage I	612 (68.8%)
Stage II	57 (6.4%)
Stage III	129 (14.5%)
Stage IV	90 (10.1%)
Stage unknown	1 (0.1%)
**Insurance**	
Private	441 (49/6%)
Medicaid	39 (4.4%)
Medicare	374 (42.1%)
Self-pay	16 (1.8%)
Other/unknown	18 (2%)
**Body mass index**	
Mean	34.6 mg/m^2^
**Distance from hospital**	
Mean	23.5 miles

In terms of insurance status, the longest treatment interval was noted in the Medicaid group with a mean treatment delay of 78 days followed by Medicare with 54 days (Table 2). The shortest interval was noted within the private insurance group with 38.4 days and was found to be clinically significant (*p* < 0.001). Even when stratified by stage of disease, Medicaid and Medicare participants continued to have longer treatment intervals (Table 3).

**Table 2:** **Mean treatment time (days) in relationship to race, insurance status, and vital status**.

Demographics	Mean treatment time (days)	*p* value
**Race**		
White	42.6	*p* = 0.048
African-American	57.3	
Hispanic	58.2	
Asian	28.6	
Other	54	
Total	47.6	
**Insurance**		
Private	38.4	*p* < 0.001
Medicaid	78.1	
Medicare	54.1	
Self-pay	53.5	
Other/unknown	63.6	
Total	47.9	
**Vital status**		
Alive	43.3	*p* < 0.001
Dead	64.8	
Total	47.9	

**Table 3:** **Mean treatment time in relation to insurance per stage**.

Insurance	Stage I	Stage II	Stage III	Stage IV

	Mean treatment interval (days)			
Private	39.0	50.8	34.4	37.3
Medicaid	53.1	275.0	40.4	179.0
Medicare	53.6	65.4	48.3	38.9
Other/unknown	109.0	46.0	40.0	3.5
Total	46.4	66.3	42.8	47.0
	*p* = 0.01	*p* = 0.06	*p* = 0.489	*p* = 0.03

Analysis of stage and insurance status as it pertains to survival demonstrated similar results (Table 4). For Stage I disease, 97.1% of patients with private insurance were alive compared to 95.8% in Medicaid and 77.1% for Medicare; 2.9% of private insurance patients were dead compared to 4.2 and 22.3%, respectively, for Medicaid and Medicare groups. Similar results were seen in Stage III disease with alive status for 72.7, 62.5, and 53.4% in private, Medicaid, and Medicare groups, respectively. In Stages II and IV, the private and Medicare patients had similar results in terms of survival. Of note, data are only available for all cause mortality.

**Table 4:** **Stage and insurance status as it pertains to survival**.

Insurance	Stage I		Stage II		Stage III		Stage IV	
	Alive	Dead	Alive	Dead	Alive	Dead	Alive	Dead
Medicaid	23 (95.8%)	1 (4.2%)	2 (100%)	0 (0%)	5 (62.5%)	5 (37.5%)	0 (0%)	5 (100%)
Medicare	171 (77.7%)	49 (22.3%)	21 (70%)	9 (30%)	39 (53.4%)	34 (46.6%)	17 (34%)	33 (66%)
No insurance	14 (93.3%)	1 (6.7%)					1 (100%)	0 (0%)
Private	331 (97.1%)	10 (2.9%)	18 (75%)	6 (25%)	32 (72.7%)	12 (27.3%)	11 (34.4%)	21 (65.6%)
Other/unknown	11 (91.7%)	1 (8.3%)	1 (100%)	0 (0%)	0 (0%)	3 (100%)	1 (50%)	1 (50%)
Total	550 (89.9%)	62 (10.1%)	42 (73.7%)	15 (26.3)	77 (59.7%)	52 (40.3%)	30 (33.3%)	60 (66.7%)

Racial differences were noted in time to treatment intervals. Caucasian women had a shorter mean treatment interval (42.6 days) as compared to African-American women (57.3 days) and Hispanics (58.2 days). The shortest treatment interval time was noted in Asian patients with 28.6 days. These differences were found to be statistically significant between groups (*p* = 0.048) (Table 2).

Of note, a multivariate analysis was performed but was felt not to show any further informative statistics. The analysis shows that three effects remain in the multiple regression analysis – a dummy code for private insurance, a dummy code for Medicare, and a dummy code for African-American race. The two insurance codes are associated with shorter intervals, being African-American is associated with longer intervals. The interval variable was transformed to better meet assumptions of normal residuals (a square root transformation). These results are in rough agreement with the univariate results, and much of the difference may be accountable to collinearity between these measures (e.g., African-American and/or Hispanic race/ethnicity with use of Medicaid).

Distance from the health center and BMI were not found to be statistically significant for a time to treatment interval.

## Discussion

In our study, patients with endometrial cancer without private insurance experienced significantly longer interval time to treatment compared to patients with private insurance. In addition, we also found increased time to treatment interval to be associated with a decreased survival. Race, BMI, and distance to treatment center were all not significantly correlated with interval treatment time. This confirms our hypothesis that socioeconomic status appears to negatively impact survival. This study supports the findings of Fedewa et al., who also found a significantly improved survival in patients with private insurance (7). The authors speculated that patients with public insurance are less likely to be managed by a gynecologic oncologist. In contrast, a gynecologic oncologist treated all the patients in our sample. Our finding of increased time to treatment interval adds another possible explanation for both Fedewa’s findings and ours. Our results mirror the greater body of literature regarding insurance disparities in cancer mortality between the underinsured and the privately insured, especially with regards to breast cancer, which has partially been attributed to decreased cancer surveillance in this population (8, 9). Interestingly, in the breast cancer literature, this disparity in surveillance persisted even in high income adults without insurance (10). Our findings did correlate with the overall body of literature on insurance status and cancer disparities and wait time (7, 11–14).

Medicaid covers a disproportionately high percentage of minorities, specifically black patients, although whites make up a higher percentage of total Medicaid beneficiaries (12). Furthermore, many studies have found race to be a significant predictor of poor outcomes despite equal insurance status and providers (15). These studies did not assess wait time, and thus, perhaps race had an effect there. Increased interval wait time is of particular concern because patients with Medicaid and without insurance are more likely to present at diagnosis with more advanced disease, and thus, this population requires timely treatment (12). A perceived inability to afford medical care could be a major contributor to advanced presentation in the uninsured (8). It is unclear, however, in our study if insurance inequality within races accounted for significance of delay in treatment and decrease survival with racial groups or if race itself was a confounder in the delay in treatment within insurance groups.

Elit et al. reported that a delay in treatment was related to a decrease in overall survival for patients with uterine cancer in a Canadian population (11). In this study, they demonstrated that a wait time of more than 12 weeks had a significantly worse survival than patients with a wait time of 2.1–6 weeks [HR 0.79 (95% CI 0.7–0.91)] and wait time 6–12 weeks [HR 0.8 (95% CI 0.71–0.91)]. They postulated that the delay in treatment may be due to centralization of uterine cancer surgical care to gynecologic oncologist at teaching hospitals and less availability of operating room times. They also state that this increase in wait times to surgery may counteract any benefit seen as a result of additional expertise from gynecologic oncology regarding surgical staging. Our study demonstrates a similar correlation between survival and time to treatment. However, all mean delay in treatment time in our study was <12 weeks (84 days), with the longest mean delay in treatment time seen being 78.1 days (11 weeks) in the Medicaid group. Our study differs from the Elit study in that it was done in a single teaching institution with patients who receive surgical care from only gynecologic oncologists; therefore, our delay in treatment cannot be justified by less availability to operating room times or delay in referral time alone. Our study was also done in a different health care system where different insurance statuses exist and not the national health care system in Canada.

There were some limitations to our study. Our population was geographically limited to one tertiary care institution in Chicago. Our sample was also not nationally representative due to its inclusion of patients seeking care at a tertiary care institution. Additionally, we could not account for patient factors, including adherence to treatment recommendations, provider preference, or comorbidities, which could have limited a definitive surgical option. We also did not account for cancer histology, which is a known prognostic indicator. Information on cause of death was not available; hence, cancer-specific deaths could not be identified.

Future studies focusing on time to treatment interval, specifically at what time to treatment interval is survival affected, are needed. Further study of the relationship of race, socioeconomic status, and time to treatment will aid providers in optimizing care in an era of increasing restriction of resources.

In conclusion, we found a significant decrease in survival with longer delay between diagnosis and treatment. In addition, this delay was directly associated with insurance status and race in our population of endometrial cancer patients treated at a large, tertiary care institution.

## Author Contributions

SD and DD wrote the manuscript, conducted the chart abstraction, and managed the project; AM and DD abstracted charts and helped write the manuscript; LF performed statistical analysis and consulting and edited the manuscript; BR, EY, JR, and AG edited the manuscript.

## Conflict of Interest Statement

The authors declare that the research was conducted in the absence of any commercial or financial relationships that could be construed as a potential conflict of interest.
